# ERRATUM: Effect of insulin-like growth factor binding protein-1 on integrin signalling and the induction of apoptosis in human breast cancer cells

**DOI:** 10.1530/JME-22-0141e

**Published:** 2025-10-03

**Authors:** C M Perks, P V Newcomb, M R Norman, J M Holly

**Affiliations:** ^1^ Division of Surgery, Department of Hospital Medicine, Bristol Royal Infirmary, Bristol, UK; ^2^ Department of Medicine, Bristol Royal Infirmary, Bristol, UK

The journal and publisher apologise for an error in the above paper, which appeared in volume 22, part 2, pages 141–150 . The error relates to Fig. 3, given on page 146, in which the image given in panel C was mistakenly replicated as panel D during journal production. The correct image for panel D had been present in the author proofs.

The original Fig. 3, as supplied by the authors, is given in full below:

**Figure 3:**
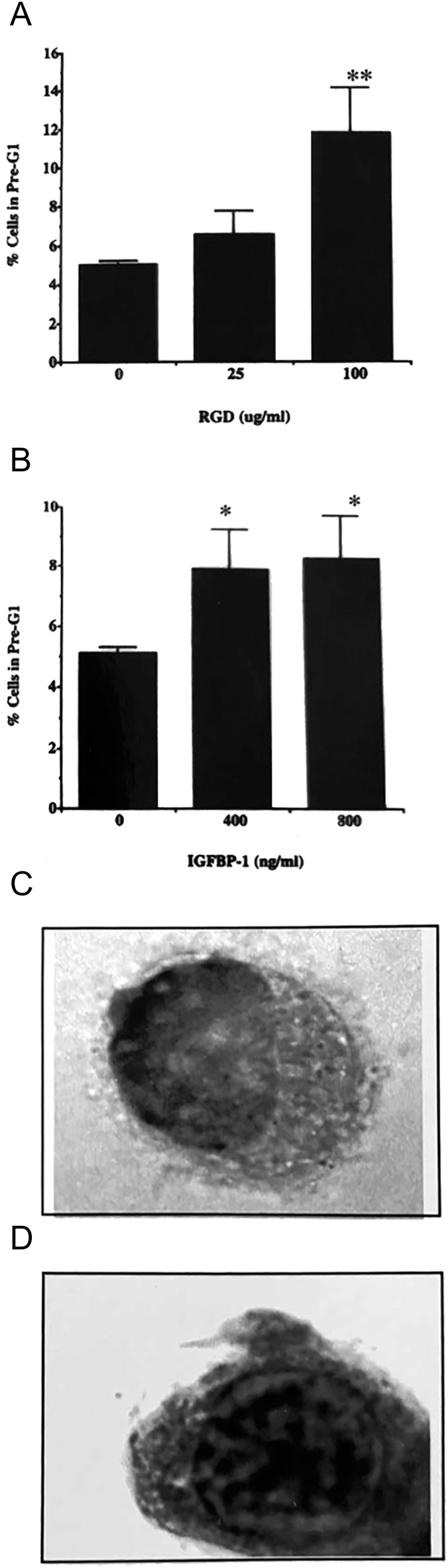
Measurement of apoptosis in T47D cells treated with a synthetic RGD-containing peptide (A) and IGFBP-1 (B) for 24 h. Cells were grown in six-well plates, allowed to settle and switched to SFM for 24 h prior to treatment. Cells were then incubated with either (A) a synthetic RGD-containing peptide (0–100 μg/mL) or (B) IGFBP-1 (0–800 ng/mL) for a further 24 h. Cells were analysed for apoptosis by flow cytometry as outlined in Materials and Methods. Results represent the mean ± SEM of three wells from experiments repeated at least three times. (C and D) Photomicrographs of control cells and those treated with 800 ng/mL IGFBP-1, respectively. Cells were cytospun and stained with Wright’s stain in an automated stainer (Lillie 1977). Photomicrographs were taken under oil immersion at a magnification of ×100.
